# Spotted fever group rickettsiae in *Dermacentor reticulatus* and *Haemaphysalis punctata* ticks in the UK

**DOI:** 10.1186/1756-3305-6-212

**Published:** 2013-07-19

**Authors:** Ellen Tijsse-Klasen, Kayleigh M Hansford, Setareh Jahfari, Paul Phipps, Hein Sprong, Jolyon M Medlock

**Affiliations:** 1 Laboratory for Zoonoses and Environmental Microbiology, National Institute for Public Health and the Environment (RIVM), Bilthoven, The Netherlands; 2 Medical Entomology & Zoonoses Ecology Group, Public Health England, Porton Down, UK; 3 Wildlife Zoonoses and Vector Borne Disease Research Group, Animal Health and Veterinaries Laboratory Agency–Weybridge, New Haw, Addlestone KT15 3NB, Surrey

**Keywords:** *Haemaphysalis*, *Rickettsia massiliae*, UK, *Dermacentor*, Rickettsiae, Ticks

## Abstract

**Background:**

Spotted fever group (SFG) rickettsiae have recently been identified for the first time in UK ticks. This included the findings of *Rickettsia helvetica* in *Ixodes ricinus* and *Rickettsia raoultii* in *Dermacentor reticulatus*. This paper further investigates the occurrence of SFG rickettsiae in additional geographically distinct populations of *D*. *reticulatus*, and for the first time, investigates the occurrence of SFG rickettsiae in UK populations of *Haemaphysalis punctata* ticks.

**Methods:**

Questing *D*. *reticulatus* and *H*. *punctata* were collected at a number of sites in England and Wales. DNA from questing ticks was extracted by alkaline lysis and detection of rickettsiae DNA was performed, in addition to detection of *A*. *phagocytophilum*, *N*. *mikurensis*, *C*. *burnetii* and *B*. *burgdorferi* sensu lato.

**Results:**

This paper builds on previous findings to include the detection of spotted fever *Rickettsia* which showed the highest homology to *Rickettsia massiliae* in *Haemaphysalis punctata*, as well as *R*. *helvetica* in *D*. *reticulatus*. The occurrence of SFG rickettsiae in *D*. *reticulatus* in the UK appears to be confined only to Welsh and Essex populations, with no evidence so far from Devon. Similarly, the occurrence of SFG rickettsiae in *H*. *punctata* appears confined to one of two farms known to be infested with this tick in North Kent, with no evidence so far from the Sussex populations. *Anaplasma phagocytophilum*, *Neoehrlichia mikurensis*, *Coxiella burnetii* and *Borrelia burgdorferi* sensu lato DNA was not detected in any of the ticks.

**Conclusion:**

These two tick species are highly restricted in their distribution in England and Wales, but where they do occur they can be abundant. Following detection of these SFG rickettsiae in additional UK tick species, as well as *I*. *ricinus*, research should now be directed towards clarifying firstly the geographic distribution of SFG rickettsiae in UK ticks, and secondly to assess the prevalence rates in ticks, wild and domesticated animals and humans to identify the drivers for disease transmission and their public health significance.

## Background

Tick-borne rickettsioses are caused by intracellular bacteria belonging to the spotted fever group (SFG) of the genus *Rickettsia*. Rickettsial diseases reported in Europe include Mediterranean spotted fever (or MSF-like illness), tick-borne lymphadenopathy (TIBOLA), *Dermacentor*-borne necrosis erythema lymphadenopathy (DEBONEL) and Lymphangitis-associated rickettsiosis (LAR) [1]. New SFG rickettsiae have been detected in recent years with the improvement of molecular techniques, and a number of these have emerged as human pathogens [2]. Although *Rickettsia conorii* (the causative agent of MSF) is the most frequently reported cause of rickettsiosis in Europe, other *Rickettsia* spp. (including *R*. *massiliae*, *R*. *monacensis* and *R*. *aeschlimannii*) cause similar clinical manifestations, and may account for some of these MSF cases [1]. There is still a lack of data on the occurrence and prevalence of SFG rickettsiae across Europe [2] and a recent study in the UK has detected, for the first time, evidence of several species of SFG rickettsia*e* in British ticks, notably *Rickettsia helvetica* in *Ixodes ricinus* and *R*. *raoultii* in *Dermacentor reticulatus*[3]. *Ixodes ricinus* is the most common tick species in the UK [4] occurring in a range of habitats [5] and is the main vector for a number of tick-borne diseases including Lyme borreliosis, louping ill virus, babesiosis and anaplasmosis. *Rickettsia helvetica* has a widespread distribution within the UK, including parts of southwest England and northern Scotland [3]. *Dermacentor reticulatus* is more restricted in its distribution and has been reported from sand dune habitats in western Wales, coastal grassland dominated by sheep in Devon [6] as well as from parts of Essex [7,8]. Evidence of *R*. *raoultii* has been reported from *D*. *reticulatus* populations from Wales and Essex. However, no sampling of the Devon *D*. *reticulatus* population has so far been carried out.

The aims of this study were to further assess the occurrence of SFG rickettsiae in populations of *D*. *reticulatus* from Devon, as well as in UK populations of *Haemaphysalis punctata*. This species, known as the red sheep tick, is limited in its distribution in the UK. Historical records in the UK are mostly associated with migratory birds surveyed at bird observatories [9] suggesting that they are frequently imported. The only historical records associated with humans or livestock are from various sites in south-east England (Essex and Kent; [9]) as well as from Aberdaron, North Wales [10]. Current established populations of this tick are known to occur at a small number of sheep-grazed farms and grassland sites in coastal North Kent and coastal East Sussex (Medlock *et al*, unpublished). This study additionally aimed to assess the occurrence of *Rickettsia sp*. in the known populations of *H*. *punctata* in southeast England.

To date, no *Rickettsia* sp. have been detected in *H*. *punctata* in the UK. The changing epidemiology of rickettsial species in Europe, and the association of *H*. *punctata* with livestock and humans (Public Health England tick recording scheme, unpublished) makes the investigation into the role of this and other British tick spp. in the transmission of SFG rickettsiae important. In an attempt to further quantify the potential risk of transmission of SFG rickettsiae and other bacterial pathogens to humans following tick bites in the UK, we investigated the presence of *Rickettsia* spp., *Anaplasma phagocytophilum*, *Neoehrlichia mikurensis*, *Coxiella burnetii* and *Borrelia burgdorferi* sensu lato in both *H*. *punctata* and *D*. *reticulatus* ticks. Most of these microorganisms are extremely difficult to culture from tick lysates and to identify using conventional microbiological tools, such as microscopy. The presence of these pathogens was therefore assessed by the detection of specific DNA-fragments using PCR-based techniques as described in the following section.

## Methods

**Figure 1:**
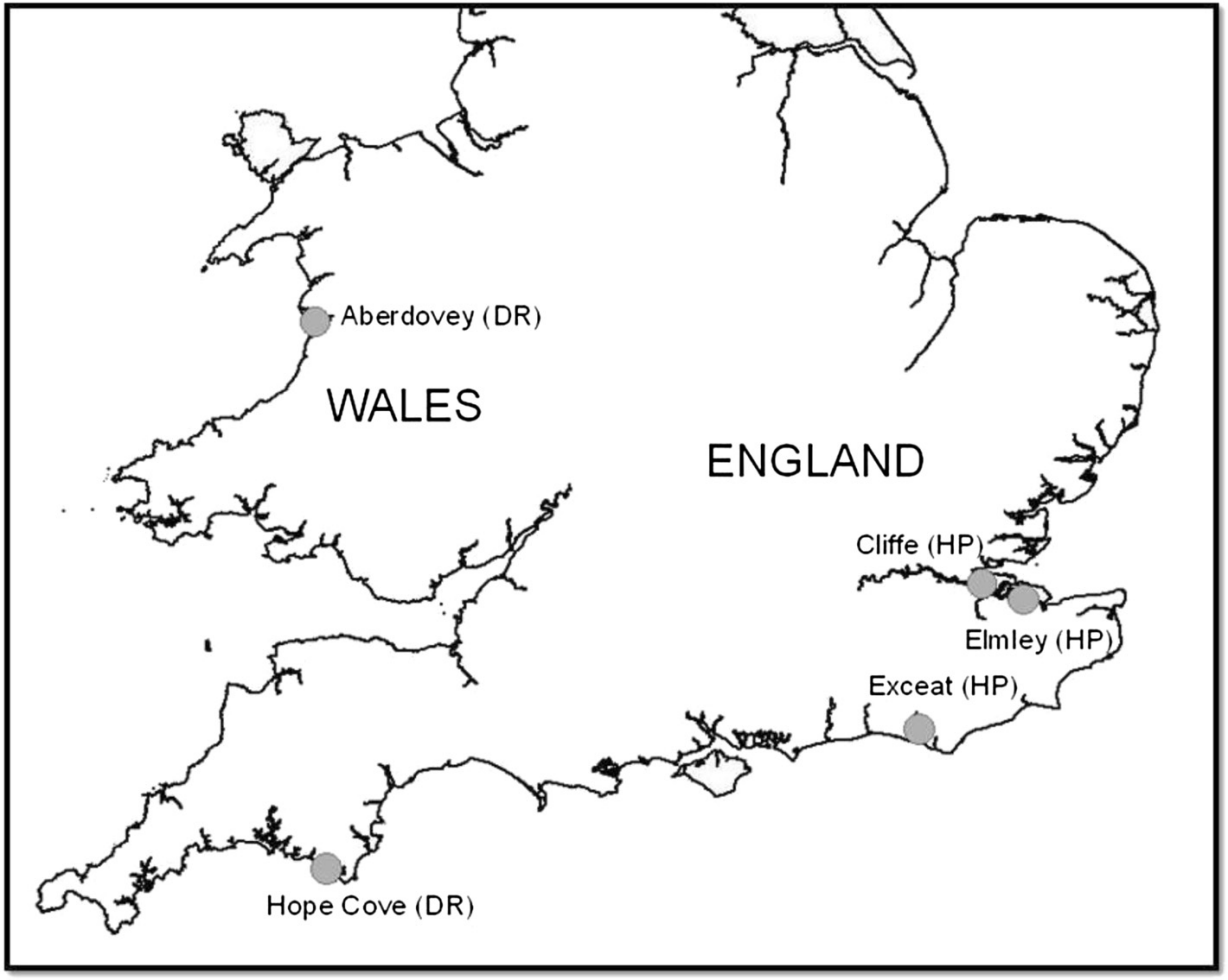
**Map of England and Wales showing locations of field sites for tick collections.** DR = *Dermacentor reticulatus*, HP = *Haemaphysalis punctata*. The map contains ordnance survey data © Crown copyright and database right 2013.

Questing *D*. *reticulatus* and *H*. *punctata* were collected by dragging a 1 × 1 m cloth over vegetation at a number of sites in England and Wales (Figure 1). For *D*. *reticulatus*, adult ticks were collected from Bolt Tail (50.2°N, 3.9°W; nr Hope Cove, Devon) on 22^nd^ March 2011 and from Aberdovey (52.5°N, 4.1°W; west Wales) on 9^th^ March 2011. The site in Wales lies 10 kilometres south of the previous sites surveyed for *D*. *reticulatus* and tested for *Rickettsia*[3]. *H*. *punctata* adults and nymphs were collected from Cooling Marshes at Cliffe (51.5°N, 0.5°E, north Kent), Elmley (51.4°N, 0.8°E, north Kent) and Seven Sisters Country Park, Exceat (50.8°N, 0.1°E, East Sussex) on 3^rd^ and 4^th^ May 2011. Ticks were stored in plastic vials during transit, frozen and stored at -80°C. Ticks were identified using published keys and sent to RIVM for testing.

DNA from questing ticks was extracted by alkaline lysis (10364588). Detection of rickettsiae DNA was performed as described previously (21087541). Briefly, a 360-basepair fragment of the 16S ribosomal RNA gene was amplified by PCR with the HotStarTaq master mix (Qiagen, Venlo, The Netherlands) using 5′-AACGCTATCGGTATGCTTAACA and 5′-ACTCACTCGGTATTGCTGGA-3 as primers with the following conditions: 15 min 94°C, then cycles of 20 s 94°C, 30 s 72°C, 30 s 72°C lowering the annealing temperature 1°C each cycle till reaching 62°C, then 40 cycles at this annealing temperature and ending by 10 min 72°C. To avoid possible contamination of the tick lysates, the positive control was a diluted DNA sample from a *R*. *africae*-positive skin biopt (21867422). The PCR products were sequenced by dideoxy-dye termination sequencing and compared with reference sequences from GenBank (http://www.ncbi.nlm.nih.gov/) after subtraction of the primer sequences. The 16S-rRNA sequences were analysed using BioNumerics 6.6 (Applied Maths, Kortrijk, Belgium). Pairwise-similarity analyses of the sequences and related organisms were conducted in BioNumerics, using the Unweighted Pair Group Method with Arithmetic Mean (UPGMA) algorithm. DNA sequences are available upon request.

Detection of A. *phagocytophilum* (22515314), *N*. *mikurensis* (22515314), *C*. *burnetii* (21824373) and *B*. *burgdorferi* sensu lato (23279105) was performed as described previously. Positive controls consisted of PCR-positive, and sequencing-confirmed *Ixodes ricinus* lysates. *Coxiella burnetii* Nine Mile RSA phase I DNA was used as a positive control in the qPCR for *C*. *burnetii* (22189106). To minimise cross contamination and false-positive results, positive and negative controls were included in each batch tested. DNA extraction, PCR master mix preparation, sample addition, and PCR analysis were performed in assigned separate labs.

## Results and discussion

**Figure 2:**
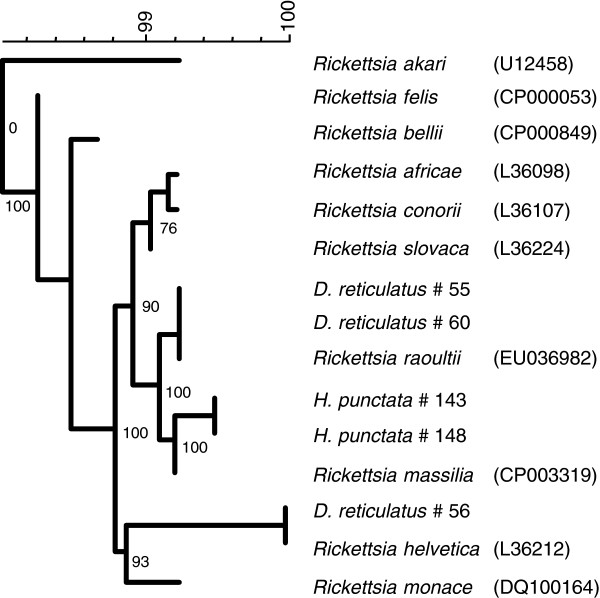
**Uncorrected neighbour joining tree of *Rickettsia *spp.isolates found in this study and several reference sequences from Genbank.** Values at branches show cophenetic correlations.

A ~360 basepair fragment of the 16S rRNA gene of *Rickettsia* species was detected in 3/61 (5%) *D*. *reticulatus* and 2/100 (2%) *H*. *punctata* tested (Table 1 and 2, Figure 2). A 338-bp fragment of one of the 16S rRNA sequences of the positive *D*. *reticulatus* showed 100% homology with *R*. *helvetica* (Genbank accession number L36212) and the other two showed 100% homology (360 bp) with *R*. *raoultii* (DQ365809). All positive *D*. *reticulatus* ticks came from the site in Wales, with no evidence of SFG rickettsiae in the Devon populations. Both 16S rRNA sequences of the positive *H*. *punctata* showed the highest homology to *R*. *massiliae* (CP003319) in GenBank, which corresponds to only one mismatch in 359 bp (99.7%). Further typing is necessary to elucidate this spotted fever rickettsia to the species level. DNA of *B*. *burgdorferi s*.*l*., *A*. *phagocytophilum*, *N*. *mikurensis* and *C*. *burnetii* was not detected in any of the 160 tick lysates.

**Table 1:** **Number**, **life**-**stage** (**F** = **females**, **M** = **male**, **N** = **nymph**) **and geographic location of*D***. ***reticulatus*tested for the presence of rickettsiae**

**Site**	***D. reticulatus***		***R. helvetica***	***R. raoultii***	**Total positive**
Aberdovey (Wales)	F	5	1	1	40%
	M	2	0	1	50%
Devon (England)	F	42	0	0	0%
	M	12	0	0	0%

**Table 2:** **Number**, **life**-**stage** (**F** = **females**, **M** = **male**, **N** = **nymph**) **and geographic location of*H***. ***punctata*tested for the presence of rickettsiae**

**Site**	***H. punctata***		***R. massiliae***	**Total positive**
Cliffe (England)	F	14	0	0%
	M	7	0	0%
	N	25	2	8%
Cuckmere haven (England)	F	19	0	0%
	M	16	0	0%
	N	9	0	0%
Elmley (England)	F	4	0	0%
	M	5	0	0%

This paper provides further evidence of the occurrence of SFG rickettsiae in British ticks. The detection of *R*. *raoultii* in an additional Welsh population of *D*. *reticulatus* is not unexpected, and infers that there is likely to have been interaction between these geographically separate tick populations through the movement of animals (possibly sheep or dogs). Furthermore, as reported previously [3] the detection of *R*. *raoultii* in populations of *D*. *reticulatus* in Essex, suggests that this new population [7] is derived from the Welsh population through movement of animals. This is supported by the absence of *R*. *raoultii* in Devon populations of *D*. *reticulatus*. In addition, the detection of *R*. *helvetica* in *D*. *reticulatus* is novel for the UK, and suggests that this tick species may now be considered as a potential vector species in addition to *I*. *ricinus*. However, so far it has only been found in *D*. *reticulatus* at one location in Wales.

Like other *Rickettsia* spp., the emergence of *R*. *massiliae* as a human pathogen was not recognised until many years after it was first isolated from *Rhipicephalus sanguineus* ticks in an area near Marseille, France in 1992 [11]. The first human case of *R*. *massiliae* infection to be reported was identified in 2005, when a sample taken from a patient hospitalised in Sicily during 1985 tested positive [12]. Two further human cases have been identified since [13,14]. *Rickettsia massiliae* causes MSF-like illness with documented cases presenting with fever, chills, malaise, rash, night sweats, necrotic eschar, maculopapular rash. Hepatomegaly [12], visual impairment [14], and convulsions [13] have also been associated with *R*. *massiliae* infection.

It has been suggested that the role of *R*. *massiliae* in causing MSF may be more important than previously realised [1]. A recent study in Spain demonstrated a higher prevalence of infection with *R*. *massiliae* compared to *R*. *conorii*, [15] and eco-epidemiological investigations into a recent cluster of MSF cases in France revealed co-infection of *R*. *conorii* and *R*. *massiliae* in *Rhipicephalus sanguineus* for the first time [16]. The presence of *R*. *massiliae* in an area together with *R*. *conorii* needs further investigation to differentiate between their individual clinical manifestations.

*Rhipicephalus sanguineus* and *Rhipicephalus turanicus* appear to be important vectors of *R*. *massiliae* and have tested positive in several European and African countries [12]. Although not confirmed as vectors, other ticks species may play a role in the transmission of *R*. *massiliae*, which has also been detected in *I*. *ricinus* ticks [2,15]. It has been suggested that *R*. *massiliae* DNA in *I*. *ricinus* ticks resulted from recent blood meals instead of successful colonization of the tick [15]. It would be of interest to continue surveillance of rickettsiae in other tick species in Britain, particularly in questing *I*. *ricinus*, which is known to be infected with a range of SFG rickettsiae and is the main UK tick species to bite humans. Furthermore, given the recent change in tick controls on travelling pets, ongoing surveillance for *R*. *sanguineus* as well as routine testing for all SFG rickettsiae will continue to be important.

## Conclusion

There is sufficient evidence that UK tick populations are changing, with a number of drivers (e.g. climatic and environmental change, movement/expansion of livestock and wild animals, changes in land use) favouring the spread of *I*. *ricinus*[4,17], as well as new findings of previously rare tick species in new locations (e.g. *D*. *reticulatus* in eastern England [6,7]), or the persistence of tick species hitherto under-recorded [6]. The potential emergence of tick-borne spotted fever group rickettsiae as human and veterinary pathogens in the UK therefore warrants further investigation. Research should now be directed towards clarifying, firstly, the geographic distribution of SFG rickettsiae in UK ticks, and secondly to assess the prevalence rates in ticks, wild and domesticated animals and humans to identify the drivers for disease transmission and their public health significance.

## Competing interests

The authors declare that they have no competing interests.

## Authors’ contributions

ETK carried out molecular genetic studies for detection and sequencing of Rickettsia’s and drafted the results. SJ carried out the molecular detection of other tick-borne pathogens. KMH conducted field research and performed the literature review, PP conducted field research and provided veterinary expertise, HS performed the phylogenetic analyses, JMM conceived the study, coordinated and conducted field research and drafted the manuscript. All authors read and approved the final version of the manuscript.
